# Modified Stage Grouping of Diffuse Large B-Cell Lymphoma Involving the Same Side of the Diaphragm in the Rituximab Era

**DOI:** 10.3389/fonc.2022.888925

**Published:** 2022-05-27

**Authors:** Hyehyun Jeong, Hyungwoo Cho, Jung Yong Hong, Dae Ho Lee, Shin Kim, Kyoungmin Lee, Eun Hee Kang, Jung Sun Park, Jin Sook Ryu, Jooryung Huh, Cheolwon Suh

**Affiliations:** ^1^Lymphoma/Myeloma Program, Department of Oncology, Asan Medical Center, University of Ulsan College of Medicine, Seoul, South Korea; ^2^Department of Nuclear Medicine, Asan Medical Center, University of Ulsan College of Medicine, Seoul, South Korea; ^3^Department of Pathology, Asan Medical Center, University of Ulsan College of Medicine, Seoul, South Korea

**Keywords:** diffuse large B-cell lymphoma, same side of diaphragm, anatomical staging, rituximab era, prognosis

## Abstract

Among patients with diffuse large B-cell lymphoma (DLBCL) involving the same side of the diaphragm, the prognostic implications of extranodal disease or its contiguity with the nodal lesion remain unclear. In this study, patients with DLBCL treated with R-CHOP whose disease was limited to the same side of the diaphragm were included. Survival was assessed by the presence, contiguity, and number of extranodal lesions. Among the 508 patients included, overall survival (OS) and progression-free survival (PFS) did not differ according to the presence of single extranodal involvement or its anatomical contiguity with the nodal lesion. However, patients with ≥2 extranodal involvement showed significantly inferior OS and PFS. We re-classified these patients into two groups: modified stage IIEe (≥2 extranodal involvement, n=92) and modified stage II (nodal or single extranodal involvement irrespective of anatomical contiguity, n=416). This modified staging showed improved prognostic performance based on the time-dependent ROC curve compared with Ann Arbor staging. In conclusion, the survival outcomes of patients with DLBCL on the same side of the diaphragm were associated with the number of extranodal lesions, but not with the contiguity of the lesions or presence of a single extranodal involvement. Based on these results, we propose a modified staging system (modified stage IIEe and II) for these patients.

## Introduction

Diffuse large B-cell lymphoma (DLBCL) is the most frequent, aggressive subtype of non-Hodgkin lymphoma (NHL), comprising 30–40% of all NHLs (1). Like many other malignant diseases, staging is one of the most important prognostic factors and is significantly associated with clinical outcomes in aggressive NHL (2). Assigning an appropriate cancer stage will help determine the optimal choice of therapy, allow an accurate prognosis for the patient and the family, and accurately stratify patients for clinical trials and quality assessment (3). The Ann Arbor staging system, the first and the backbone of current anatomical staging system for NHL, is used to classify patients according to the extent of disease involvement and is implemented in the Lugano staging system (4, 5). According to Ann Arbor staging, stage 2 is defined as involvement of 2 or more lymph node regions on the same side of the diaphragm. If there is involvement of a single extranodal site contiguous to the involved lymph nodes, it is referred to as stage 2 extranodal lymphoma.

However, gray areas with ambiguous definitions exist among patients whose disease extent is limited to the same side of the diaphragm in terms of accurate staging and prognoses according to it. For example, between patients with stage 2 extranodal and stage 4 lymphoma limited to the same side of the diaphragm, “more extensive extranodal disease” is designated as stage 4 lymphoma based on the Ann Arbor staging system with Cotswolds modifications (6). In the Lugano classification, “limited contiguous” extranodal involvement is designated as stage 2 extranodal lymphoma, whereas “additional non-contiguous” extra lymphatic involvement is designated as stage 4 lymphoma (5). These definitions allow for uncertainties when determining the extensiveness or contiguity of the disease. It remains unclear whether the prognoses of patients differ by anatomical contiguity of the disease on the same side of the diaphragm or whether the presence of extranodal disease affects the clinical outcomes in these patients, especially in the rituximab era (7, 8).

In this study, we assessed the prognostic implication of anatomical contiguity and the presence of extranodal disease among patients with newly diagnosed DLBCL limited to the same side of the diaphragm and suggested a modified staging system that could reflect the prognoses of these patients in the rituximab era.

## Materials and Methods

### Patients

Patients with newly diagnosed DLBCL treated with rituximab combined with cyclophosphamide, doxorubicin, vincristine, and prednisone (R-CHOP) or R-CHOP-like regimens between January 2003 and December 2018 at the Asan Medical Center were identified retrospectively. Among them, patients whose pathologic diagnoses were other than DLBCL-not otherwise specified (NOS), with Ann Arbor stage 1 disease, and whose disease extent involved both sides of the diaphragm were excluded. The remaining patients whose disease extent was limited to the same side of the diaphragm were included in this study.

This study was approved by the institutional review board of the Asan Medical Center and performed in accordance with the ethical standards of the institutional research committee and the Declaration of Helsinki. The requirement for obtaining informed consent was waived due to the retrospective nature of the study.

### Assessment and the Modified Staging System

To evaluate the prognostic implications of anatomic contiguity and the presence of extranodal disease in the study population, the patients were classified into the following four groups according to disease stage: those with 1) stage II disease, nodal stage 2; 2) stage IIEc disease, 1 extranodal involvement with contiguous nodal involvement; 3) stage IIEn disease, 1 extranodal involvement with non-contiguous nodal involvement; and 4) stage IIEe disease, ≥2 extranodal involvement but limited to the same side diaphragm. Here, lowercase c and n indicate contiguous and non-contiguous diseases, respectively. The suffix E indicates the presence of one extranodal disease and Ee indicates the presence of ≥2 extranodal diseases. The involvement of Waldeyer’s ring, thymus, and spleen was considered nodal. During the study period, ^18^fluoro-2-deoxyglucose-positron emission tomography/computed tomography (^18^FDG-PET/CT) was included as a routine staging workup. In this study, the Ann Arbor staging was written in Arabic numbers (stages 1–4), whereas the modified staging system was written in Roman numbers (stages I–IV).

### Statistical Analysis

Overall survival (OS) was defined as the time between the date of diagnosis to the date of death from any cause. Progression-free survival (PFS) was defined as the time between the date of diagnosis to the date of disease progression or death from any cause, whichever occurred first. The Kaplan–Meier method was used to estimate the survival outcomes and log-rank tests were used for comparisons. Univariate and multivariate analyses of PFS and OS were performed using the Cox proportional hazards model. Prognostic performances of the modified staging system and the Ann Arbor staging system were assessed using a time-dependent receiver operating characteristic (ROC) curve. A two-sided *p-*value of < 0.05 was considered significant. All statistical analyses were performed using R version 4.0.3 (R Foundation for Statistical Computing, Vienna, Austria).

## Results

### Patients

**Figure 1:**
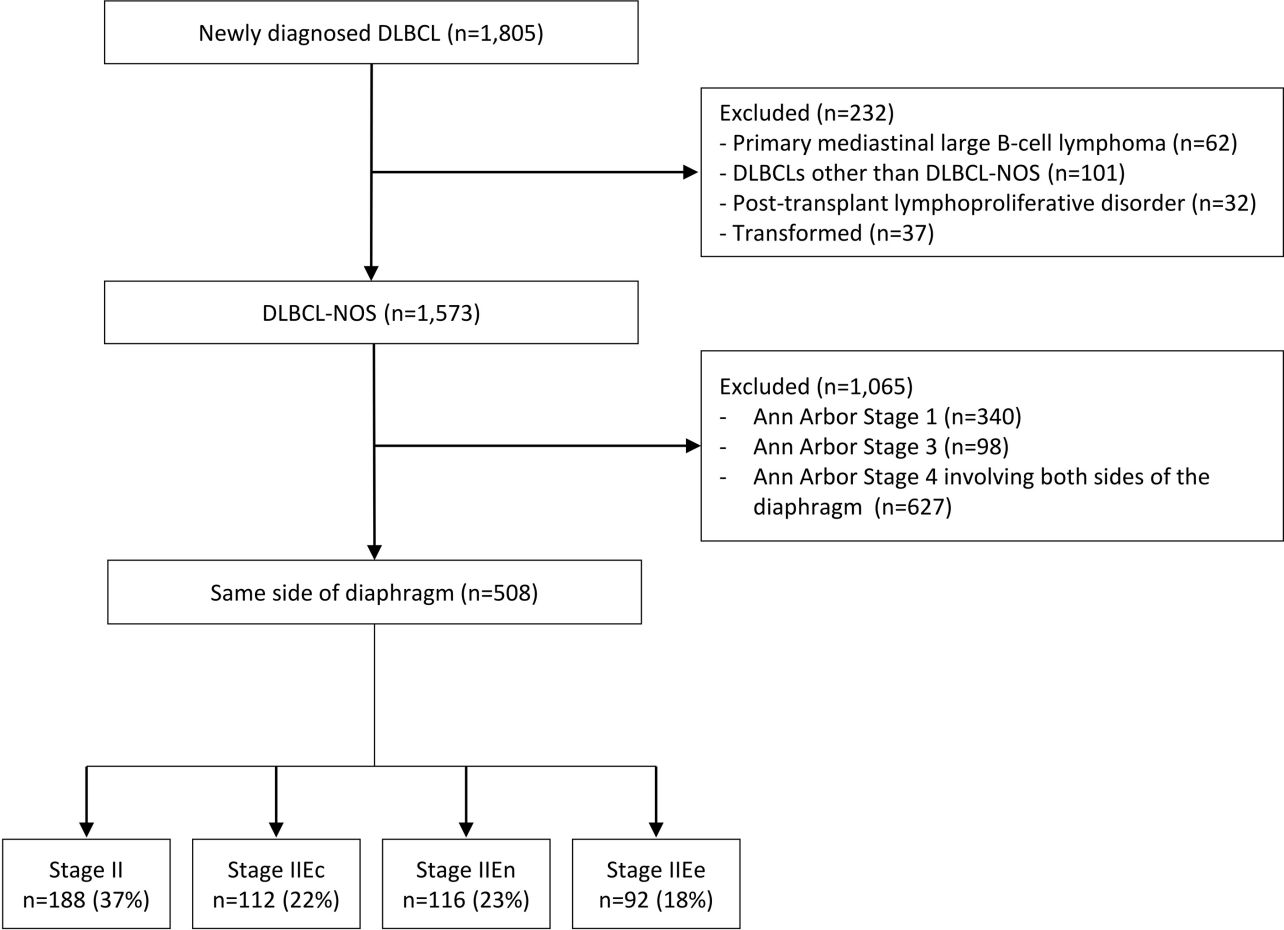
Flow diagram. DLBCL, diffuse large B-cell lymphoma; NOS, not otherwise specified. The disease stages are defined as follows: 1) II, nodal stage II, 2) IIEc, 1 extranodal involvement with contiguous nodal involvement; 3) IIEn, 1 extranodal involvement with non-contiguous nodal involvement; and 4) IIEe, ≥2 extranodal involvement but limited to the same side diaphragm.

During the study period, 1805 patients with newly diagnosed DLBCL who were treated with R-CHOP or R-CHOP-like regimens were retrospectively identified, of whom 1573 had a histological diagnosis of DLBCL-NOS. Among them, 508 patients with disease located on the same side of the diaphragm and who were not classified as Ann Arbor stage 1 were included in the analysis. Patients were divided into four groups (stage II [n = 188, 37.0%], stage IIEc [n = 112, 22.0%], stage IIEn [n = 116, 22.8%], and stage IIEe [n = 92, 18.1%]) according to the location and number of extranodal site involvements as described previously (**Figure 1**). The baseline patient characteristics by stage are shown in **Table 1**. The median age at diagnosis of all patients was 57 years (range: 16–88), which did not differ between groups. Overall, patients with IIEe disease presented with a higher international prognostic index (IPI) score (proportions of patients with higher IPI scores: overall, 5.6% [n = 28/496]; stage IIEe, 27.0% [n = 24/89], *p* < 0.001 for all stages) and bulky disease (overall, 10.8% [n = 55/508]; stage IIEe, 21.7% [n = 20/92], *p* < 0.001 for all stages).

**Table 1:** Baseline characteristics.

Characteristics	Total, n (%)	Stage II, n (%)	Stage IIEc, n (%)	Stage IIEn, n (%)	Stage IIEe, n (%)	*p*
Number, n	508 (100.0)	188 (37.0)	112 (22.0)	116 (22.8)	92 (18.1)	
Median age (range), year	57 (16–88)	57 (20–88)	56 (16–80)	59 (17–84)	60 (20–85)	0.418
Sex						0.181
Male	296 (58.3)	118 (62.8)	68 (60.7)	64 (55.2)	46 (50.0)	
Female	212 (41.7)	70 (37.2)	44 (39.3)	52 (44.8)	46 (50.0)	
Location						<0.001
Above diaphragm	252 (49.6)	159 (84.6)	23 (20.5)	45 (38.8)	25 (27.2)	
Below diaphragm	256 (50.4)	29 (15.4)	89 (79.5)	71 (61.2)	67 (72.8)	
ECOG PS						<0.001
0-1	480 (94.5)	186 (98.9)	109 (97.3)	111 (95.7)	74 (80.4)	
≥2	28 (5.5)	2 (1.1)	3 (2.7)	5 (4.3)	18 (19.6)	
Serum LDH	n = 496	n = 184	n = 107	n = 116	n = 89	<0.001
Not elevated	330 (66.5)	143 (77.7)	85 (79.4)	65 (56.0)	37 (41.6)	
Elevated	166 (33.5)	41 (22.3)	22 (20.6)	51 (44.0)	52 (58.4)	
IPI score	n = 496	n = 184	n = 107	n = 116	n = 89	<0.001
Low	336 (67.7)	168 (91.3)	91 (85.0)	64 (55.2)	13 (14.6)	
Intermediate	132 (26.6)	16 (8.7)	15 (14.0)	49 (42.2)	52 (58.4)	
High	28 (5.6)	0	1 (0.9)	3 (2.6)	24 (27.0)	
Bulky disease (≥7.5 cm)	55 (10.8)	12 (6.4)	7 (6.2)	16 (13.8)	20 (21.7)	<0.001
B symptoms	50 (9.8)	11 (5.9)	10 (8.9)	15 (12.9)	14 (15.2)	0.052
Cell of origin						0.149
Germinal center	127 (25.0)	46 (24.5)	28 (25.0)	26 (22.4)	27 (29.3)	
Non-germinal center	310 (61.0)	106 (56.4)	75 (67.0)	75 (64.7)	54 (58.7)	
Unknown	71 (14.0)	36 (19.1)	9 (8.0)	15 (12.9)	11 (12.0)	
Radiotherapy	92 (18.1)	44 (23.4)	12 (10.7)	17 (14.7)	19 (20.7)	0.029
First response	n = 487	n = 185	n = 108	n = 113	n = 81	0.009
Complete response	439 (90.1)	172 (93.0)	97 (89.8)	105 (92.9)	65 (80.2)	

ECOG PS, Eastern Cooperative Oncology Group performance status; IPI, international prognostic index; LDH, lactate dehydrogenase.

### Survival Outcomes by Contiguity and Extranodal Involvement

**Figure 2:**
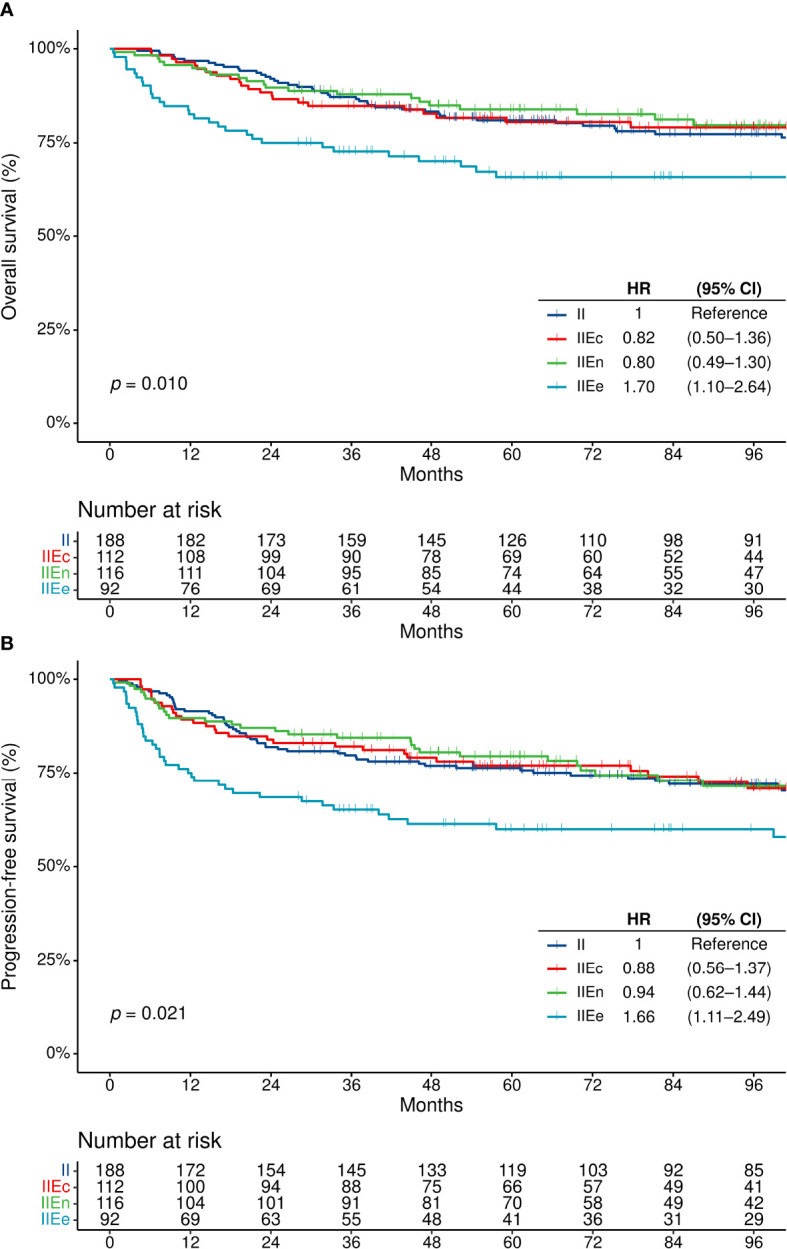
Survival outcomes in patients with disease on the same side of the diaphragm. **(A)** Overall survival, and **(B)** progression-free survival. CI, confidence interval; HR, hazard ratio; OS, overall survival; PFS, progression-free survival.

The median follow-up duration was 104.3 months (95% confidence interval [CI], 95.9–111.4). When the four groups of patients were compared, the OS and PFS did not differ between patients with stage II–IIEn disease, regardless of the presence of single extranodal involvement [stage II vs. IIEc or IIEn] or contiguity of the extranodal lesion with a nodal lesion [IIEc vs. IIEn] (**Supplementary Table S1**). However, patients with IIEe disease showed a significantly poorer OS and PFS compared with others (**Figures 2A, B**). The rates of complete remission after first-line chemotherapy were also lower in patients with stage IIEe disease (overall, 90.1% [n = 439/487]; stage IIEe, 80.2% [n = 65/81], *p* = 0.009) (**Table 1**).

### Modified Staging System

**Figure 3:**
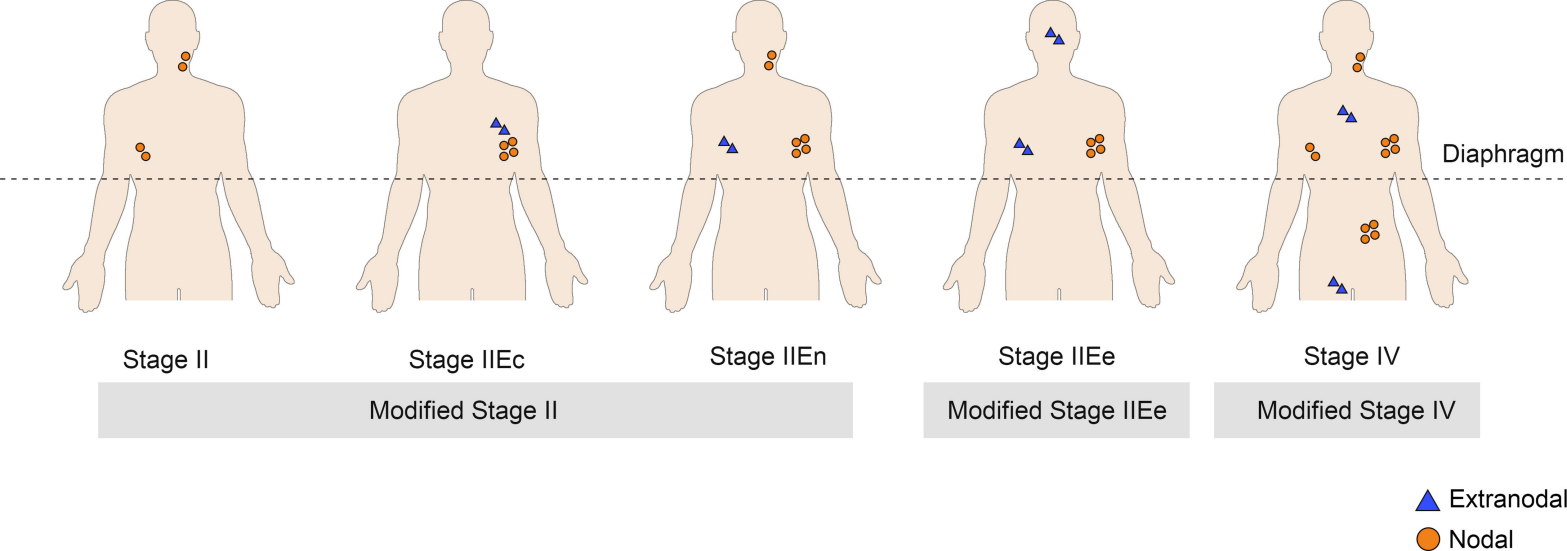
Graphic summarization of the modified staging system.

Based on the significantly poor prognoses of stage IIEe patients, the study population was further divided into the following two subgroups: 1) modified stage II (0–1 extranodal lesions on the same side of the diaphragm, regardless of the contiguity of the extranodal lesion with the nodal lesion, which included stages II, IIEc, or IIEn) (n = 416, 81.9%) and 2) modified stage IIEe (≥2 extranodal lesions on the same side of the diaphragm) (n = 92, 18.1%) (**Figure 3** and **Supplementary Table S2**).

### Prognosis of Stage IIEe Patients Using the Modified Staging System

To compare the survival outcomes of patients with stage IIEe disease with those whose diseases involved both sides of the diaphragm, additional survival analyses were performed for 1135 patients, which were the original study population (n = 508) and patients with Ann Arbor stage 4 disease involving both sides of the diaphragm (n = 627) (**Figure 1**). By Ann Arbor staging, these patients were either stage 2 (n = 369 [32.5%]) or 4 (n = 766 [67.5%]). They were regrouped into modified stage II (n = 416 [36.6%]), modified stage IIEe (n = 92 [8.1%]), and modified stage IV disease (≥ 1 extranodal lesion in both sides of the diaphragm; n = 627 [55.2%]).

**Figure 4:**
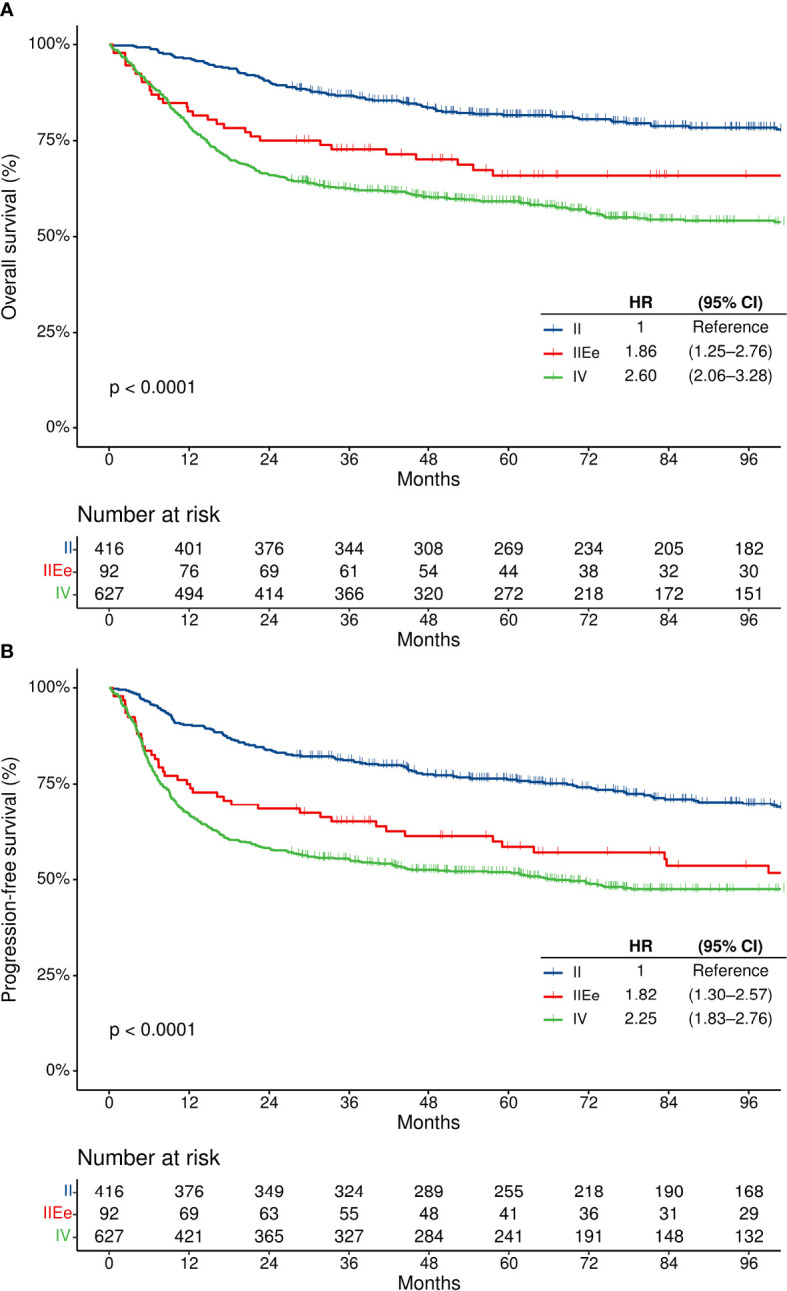
Survival outcomes according to the modified staging system. **(A)** Overall survival, and **(B)** progression-free survival. CI, confidence interval; HR, hazard ratio; OS, overall survival; PFS, progression-free survival.

Patients with modified stage IIEe disease had higher rates of IPI-high tumors than those with modified stage II but lower rates than those with modified stage IV disease (1.0% [n = 4/407] vs. 27.0% [n = 24/89] vs. 36.7% [n = 227/618] in modified stage II, IIEe, and IV disease, respectively) (**Supplementary Table S2**). There were significant differences in the OS and PFS according to the modified staging system (**Figure 4**). The two-year OS rates of the modified stage II, IIEe, and IV patients were 90.4% (95% CI, 87.1–92.9%), 75.0% (95% CI, 64.8–82.6%), and 66.0% (95% CI, 62.2–69.6%), respectively (log-rank *p*<0.001). Similarly, the two-year PFS rates of the modified stage II, IIEe, and IV patients were 83.9% (95% CI, 80.0–87.1%), 68.5% (95% CI, 57.9–76.9%), and 58.2% (95% CI, 54.3–62.0%), respectively (log-rank *p*<0.001). In the multivariate Cox regression analyses adjusted for age, performance status, and serum lactate dehydrogenase level, with modified stage II as a reference, modified stage IIEe and IV had hazard ratios of 1.36 (95% CI, 0.95–1.96) and 2.23 (95% CI, 1.75–2.84), respectively, for PFS, and 1.30 (95% CI, 0.85–1.99) and 1.73 (1.34–2.23), respectively, for OS (**Table 2**).

**Table 2:** Univariate and multivariate prognostic analyses.

	Univariate		Multivariate	
	HR (95% CI)	*p*	HR (95% CI)	*p*
**PFS**				
Age >60 years	2.34 (1.95-2.81)	<0.001	2.22 (1.84-2.68)	<0.001
Female sex	0.89 (0.74-1.06)	0.189	Not included	
ECOG PS ≥2	2.04 (1.71-2.44)	<0.001	1.45 (1.12-1.87)	0.005
Elevated serum LDH	2.82 (2.31-3.42)	<0.001	2.19 (1.77-2.71)	<0.001
Modified stage				
II	Reference		Reference	
IIEe	1.82 (1.30-2.57)	<0.001	1.36 (0.95-1.96)	0.098
IV	2.25 (1.83-2.76)	<0.001	1.56 (1.24-1.95)	<0.001
**OS**				
Age >60 years	2.80 (2.28-3.44)	<0.001	2.61 (2.11-3.23)	<0.001
Female sex	0.93 (0.77-1.14)	0.496	Not included	
ECOG PS ≥2	2.19 (1.8-2.66)	<0.001	1.66 (1.26-2.17)	<0.001
Elevated serum LDH	3.08 (2.47-3.86)	<0.001	2.23 (1.75-2.84)	<0.001
Modified stage		<0.001		
II	Reference		Reference	
IIEe	1.86 (1.25-2.76)	0.002	1.30 (0.85-1.99)	0.231
IV	2.60 (2.06-3.28)	<0.001	1.73 (1.34-2.23)	<0.001

CI, confidence interval; ECOG PS, Eastern Cooperative Oncology Group performance status; HR, hazard ratio; LDH, lactate dehydrogenase; OS, overall survival; PFS, progression-free survival.

The time-dependent areas under the receiver operating characteristic curves (AUC) for 2-year survival were 0.634 and 0.657, whereas the time-dependent AUC for 2-year progression-free survival were 0.615 and 0.642 for the Ann Arbor staging system and the modified staging system, respectively (**Supplementary Figure S3**).

## Discussion

In this study, we analyzed the prognosis of DLBCL patients with disease extent limited to the same side of the diaphragm. As a result, no differences were observed in the survival outcomes between patients with nodal disease (II) and those with single extranodal involvement (IIEc and IIEn). No significant differences were also observed in the survival outcomes of patients with single extranodal involvement in terms of the contiguity of the extranodal lesion with the nodal lesion (IIEc vs. IIEn). However, patients with ≥2 extranodal involvement (IIEe) had significantly worse OS and PFS compared with others. Based on these findings, the patients were re-classified into those with modified stage II disease (same side of the diaphragm with 0–1 extranodal involvement) and those with modified IIEe disease (same side of the diaphragm with ≥2 extranodal involvement). The new group of modified stage IIEe disease patients showed poorer survival outcomes compared with those with modified stage II disease, but better outcomes compared with those with modified stage IV disease (whose disease involved both sides of the diaphragm and at least one extranodal lesion). In addition, the modified staging system showed a higher AUC for survival prediction compared with the Ann Arbor staging system.

Previous retrospective studies reported poorer survival outcomes of extranodal disease compared with nodal disease among patients with stage 1 DLBCL or in the overall DLBCL population (9–11). However, few studies have reported the prognostic implication of extranodal disease among patients with Ann Arbor stage 2 DLBCL or patients with disease involving the same side of the diaphragm. In this study, no survival differences were observed between patients with nodal disease (Ann Arbor stage 2) and those with single extranodal disease (Ann Arbor stage 2 extranodal) in DLBCL on the same side of the diaphragm.

Prognoses by the anatomic contiguity of a single extranodal lesion with nodal lesions have rarely been reported. Based on the current Lugano staging system, staging differs according to the anatomic contiguity of the single extranodal lesion with the nodal lesion (if contiguous, stage 2; if non-contiguous, stage 4) (5). However, in this study, no survival differences were observed according to the contiguity of the single extranodal lesion with the nodal lesion (regarded as stage IIEc vs. IIEn).

Taking these findings into consideration, among patients whose disease extent was limited to the same side of the diaphragm but were not classified as stage 1 DLBCL, the following subgroups were classified as “modified stage II” disease: 1) patients with nodal lesions only, 2) patients with single extranodal lesion that was contiguous to the nodal lesion, and 3) patients with single extranodal lesion that was non-contiguous to the nodal lesion. In contrast, patients with ≥2 extranodal involvement showed significantly inferior PFS and OS compared with the modified stage II DLBCL patients in this study, which was consistent with previous studies (2, 9). These results suggest that the amount of extranodal involvement, rather than the presence of extranodal involvement itself or the anatomic contiguity of the lesion, is a more important prognostic factor for patients with DLBCL on the same side of the diaphragm. These patients were classified as having “modified stage IIEe” disease.

The survival outcomes of patients with modified stage IIEe were compared further with those of patients with modified stage IV disease involving both sides of the diaphragm. Although these patients were classified as the same stage (stage 4) according to the Ann Arbor staging system, patients with modified stage IIEe disease showed better PFS and OS than those with modified stage IV disease involving both sides of the diaphragm. Multivariate analyses showed a consistent tendency, although this was not statistically significant. The lack of statistical significance might be attributed to the small number of stage IIEe cases. This finding is in line with a recent report by Cottereau et al. who reported that the furthest distance between lesions measured by FDG-PET was associated with poorer survival in chemoimmunotherapy-treated advanced-stage DLBCL (12). Although more highly powered studies are warranted to confirm these findings, these results suggest that the distance and extent of anatomic distribution of the disease might be associated with survival outcomes.

This study was limited by its single-centered, retrospective nature. However, to the best of our knowledge, this is the first study to assess the prognostic implications of nodal/extranodal disease status, contiguity of the disease extent, and the number of extranodal involvement among patients with DLBCL on the same side of the diaphragm. All of these factors are important in clinical practice. One of the strengths of our study is the inclusion of a large number of patients who were homogeneously treated with modern chemoimmunotherapy regimens containing rituximab. Although recent molecular studies on DLBCL have opened a new horizon for the prognostication of DLBCL (13), anatomical staging based on the Ann Arbor staging system, is still widely used in clinical practice for the risk assessment of newly diagnosed DLBCL. However, as current anatomical staging was established in the pre-rituximab era and the implementation of rituximab has significantly improved the clinical outcome of patients with DLBCL (14, 15), it is necessary to re-evaluate the prognostic implications of the anatomic distribution in the rituximab era. Many previous studies sought to validate or modify risk stratification models for DLBCL such as the international prognostic index in the rituximab era (16, 17). However, the validity of anatomical staging, which comprises a backbone of such models has been rarely re-evaluated, especially regarding the gray areas of the anatomical staging as we did in this work. We believe that our modified staging system may help select the appropriate treatment strategy based on the clinical risk in the rituximab era.

In conclusion, in patients with DLBCL on the same side of the diaphragm, single extranodal involvement or the anatomic contiguity of the single extranodal lesion were not significant prognostic factors, whereas the presence of multiple extranodal disease was a poor prognostic factor. Furthermore, the survival outcome of patients with multiple extranodal disease on the same side of the diaphragm was better than that of patients with disease involving both sides of the diaphragm. Therefore, the following modified staging can be considered: 1) stage IIEe (if they have more than 1 extranodal involvement) and 2) stage II (for others irrespective of the presence of single extranodal involvement or contiguity of the involved sites if they are located on the same side of the diaphragm).

## Data Availability Statement

The datasets generated during and/or analysed during the current study are available from the corresponding author on reasonable request.

## Ethics Statement

The studies involving human participants were reviewed and approved by Institutional Review Board of Asan Medical Center. Written informed consent for participation was not required for this study in accordance with the national legislation and the institutional requirements.

## Author Contributions

HJ and HC: formal analysis, investigation, writing - original draft, visualization; JYH, DHL, JSR, and JH: resources, writing - review and editing; SK, KL, EHK, and JSP: investigation; CS: conceptualization, methodology, resources, writing - review and editing, and supervision. All authors contributed to the article and approved the submitted version.

## Conflict of Interest

The authors declare that the research was conducted in the absence of any commercial or financial relationships that could be construed as a potential conflict of interest.

## Publisher’s Note

All claims expressed in this article are solely those of the authors and do not necessarily represent those of their affiliated organizations, or those of the publisher, the editors and the reviewers. Any product that may be evaluated in this article, or claim that may be made by its manufacturer, is not guaranteed or endorsed by the publisher.
