# Supplementary material for: Modified Stage Grouping of Diffuse Large B-Cell Lymphoma Involving the Same Side of the Diaphragm in the Rituximab Era
Source: Front Oncol. 2022 May 27;12:888925. doi: 10.3389/fonc.2022.888925 (PMC9197215; doi:10.3389/fonc.2022.888925)

Supplementary Material

# Supplementary Figures and Tables

## Supplementary Table S1. Pairwise log-rank p values among the study population

| **Overall survival** |  |  |  |
| --- | --- | --- | --- |
|  | II | IIEc | IIEn |
| IIEc | 0.472 | - | - |
| IIEn | 0.386 | 0.942 | - |
| IIEe | *0.017* | *0.007* | *0.005* |
| **Progression-free survival** |  |  |  |
|  | II | IIEc | IIEn |
| IIEc | 0.559 |  |  |
| IIEn | 0.827 | 0.791 |  |
| IIEe | *0.015* | *0.008* | *0.018* |

## Supplementary Table S2. Baseline characteristics of patients with modified stage II, IIEe, and IV disease

|  | **All** | **Modified II** | **Modified IIEe** | **Modified IV** | ***p* value** |
| --- | --- | --- | --- | --- | --- |
|  | **(N=1,135)** | **(N=416)** | **(N=92)** | **(N=627)** |  |
| Age, median (range) | 60 (16–91) | 57 (16-88) | 60 (20–85) | 62 (16–91) | <0.001 |
| Sex |  |  |  |  | 0.148 |
| Male | 646 (56.9%) | 250 (60.1%) | 46 (50.0%) | 350 (55.8%) |  |
| Female | 489 (43.1%) | 166 (39.9%) | 46 (50.0%) | 277 (44.2%) |  |
| ECOG PS | N=1,134 |  |  | N=626 | <0.001 |
| 0–1 | 1,014 (89.4%) | 406 (97.6%) | 74 (80.4%) | 534 (85.3%) |  |
| ≥2 | 120 (10.6%) | 10 (2.4%) | 18 (19.6%) | 92 (14.7%) |  |
| LDH | N=1,114 | N=407 | N=89 | N=618 | <0.001 |
| Within normal | 518 (46.5%) | 293 (72.0%) | 37 (41.6%) | 188 (30.4%) |  |
| Above normal | 596 (53.5%) | 114 (28.0%) | 52 (58.4%) | 430 (69.6%) |  |
| IPI at diagnosis | N=1,114 | N=407 | N=89 | N=618 | <0.001 |
| Low | 385 (34.6%) | 323 (79.4%) | 13 (14.6%) | 49 (7.9%) |  |
| Intermediate | 474 (42.5%) | 80 (19.7%) | 52 (58.4%) | 342 (55.3%) |  |
| High | 255 (22.9%) | 4 (1.0%) | 24 (27.0%) | 227 (36.7%) |  |
| Bulky disease |  |  |  |  | 0.001 |
| No | 1,016 (89.5%) | 381 (91.6%) | 72 (78.3%) | 563 (89.8%) |  |
| Yes | 119 (10.5%) | 35 (8.4%) | 20 (21.7%) | 64 (10.2%) |  |
| B symptoms |  |  |  |  | <0.001 |
| No | 943 (83.1%) | 380 (91.3%) | 78 (84.8%) | 485 (77.4%) |  |
| Yes | 192 (16.9%) | 36 (8.7%) | 14 (15.2%) | 142 (22.6%) |  |
| Cell of origin |  |  |  |  | 0.108 |
| GCB like | 326 (28.7%) | 100 (24.0%) | 27 (29.3%) | 199 (31.7%) |  |
| non-GCB like | 663 (58.4%) | 256 (61.5%) | 54 (58.7%) | 353 (56.3%) |  |
| Unknown | 146 (12.9%) | 60 (14.4%) | 11 (12.0%) | 75 (12.0%) |  |
| Radiotherapy |  |  |  |  | 0.002 |
| No | 974 (85.8%) | 343 (82.5%) | 73 (79.3%) | 558 (89.0%) |  |
| Yes | 161 (14.2%) | 73 (17.5%) | 19 (20.7%) | 69 (11.0%) |  |

## Supplementary Figure S3. Time-dependent ROC curve based on the Ann Arbor stage and modified stage for (A) 2-year survival, and (B) 2-year progression-free survival


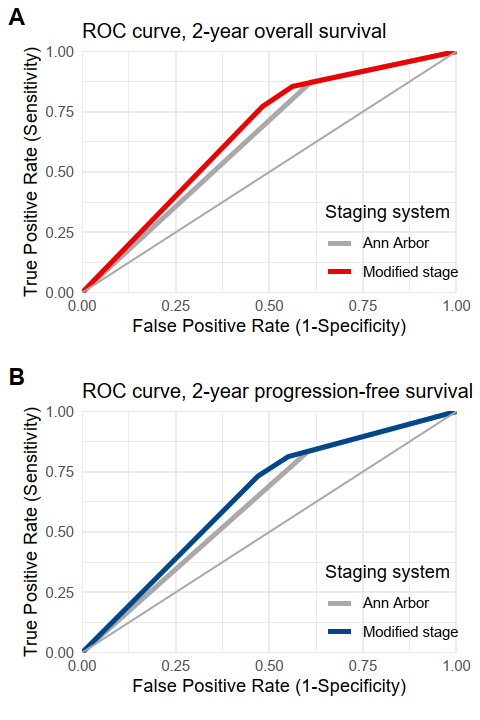

Supplement: Supplementary file 1 [file DataSheet_1.docx]
